# Sociodemographic differences in patterns of nicotine and cannabis vaping among US adults

**DOI:** 10.1016/j.pmedr.2022.101715

**Published:** 2022-01-27

**Authors:** Delvon T. Mattingly, Akash Patel, Jana L. Hirschtick, Nancy L. Fleischer

**Affiliations:** University of Michigan School of Public Health, Department of Epidemiology, Center for Social Epidemiology and Population Health, Ann Arbor, MI 48109, USA

**Keywords:** E-cigarette, Nicotine, Tobacco, Marijuana, Cannabis, Co-use, Vaping, Disparities

## Abstract

Nicotine and cannabis vaping has increased over the past several years. While patterns of cigarette and cannabis co-use are well-documented, less is known about the intersection between nicotine and cannabis vaping, especially among adults. Thus, we categorized nicotine and cannabis vaping among adults (18+) who currently (past 30-day) used electronic vapor products (EVPs) from Wave 4 of the Population Assessment of Tobacco and Health Study (n = 3795) as: 1) nicotine only, 2) cannabis only, 3) nicotine and cannabis, and 4) non-nicotine/non-cannabis e-liquid. We calculated vaping pattern proportions overall and by sociodemographic characteristics. Adjusted multinomial logistic regression models assessed associations between sociodemographic characteristics and vaping categories relative to nicotine-only vaping. Approximately half (54.2%) of adults who currently used EVPs vaped nicotine only, 7.4% vaped cannabis only, 23.8% vaped nicotine and cannabis, and 14.6% vaped non-nicotine/non-cannabis e-liquid. Young adults (aged 18–24) (vs. adults aged 35+) had at least three-fold greater odds of vaping cannabis only, nicotine and cannabis, and non-nicotine/non-cannabis e-liquid, compared to nicotine only. Hispanic and non-Hispanic Black (vs. non-Hispanic White) adults had 2.5–3 times greater odds of vaping cannabis only and non-nicotine/non-cannabis e-liquid, compared to nicotine only. Sexual minority adults (vs. heterosexual adults) had 1.5 times greater odds of vaping nicotine and cannabis, compared to nicotine only. Nearly half of adults who vaped EVPs consumed something other than nicotine only, and nicotine/cannabis vaping patterns differed by sociodemographic groups. Vaping and nicotine reduction efforts must recognize that adults who currently vape may be vaping cannabis, or neither nicotine nor cannabis.

## Highlights

• About half (54.2%) of adults who used electronic vapor products (EVPs) vaped nicotine only.
• Only 7.4% of adults who used EVPs vaped cannabis only, while one-fourth (23.8%) vaped nicotine and cannabis.
• Patterns of nicotine and cannabis vaping differed by age, race/ethnicity, education, and sexual orientation.
• Vaping nicotine and cannabis and cannabis only was more common among young adults.
• Vaping cannabis only was more common among Hispanic and non-Hispanic Black adults.

## 1 Introduction

Vaping is a public health concern. Its definition has evolved over time to include the use of electronic vapor products (EVPs) that can be used to vape nicotine and cannabis (Fataar and Hammond, 2019, Miech et al., 2017). EVP use has increased among youth and young adults since 2011 in the US (Dai and Leventhal, 2019, Wang et al., 2018). This increase has occurred alongside a surge in EVP variety and availability across the tobacco market (Barrington-Trimis et al., 2018). Concomitantly, more US states have legalized recreational and/or medical cannabis use in the past decade, possibly leading to more use and an increase in alternative methods of cannabis consumption, including vaping (Borodovsky et al., 2016, Paschall et al., 2021, Patrick et al., 2020). Evidence suggests that cannabis vaping has become more common in recent years among youth, with potential implications for cannabis vaping among adults (Miech et al., 2019, Patrick et al., 2020). However, current research examining patterns of nicotine and cannabis vaping among adults is limited.

Given that disproportionately higher rates of cannabis smoking occur among adults who use cigarettes (Rabin and George, 2015), cannabis vaping may also be common among people who vape nicotine (Fataar and Hammond, 2019, Meier and Hatsukami, 2016). While nicotine remains the most vaped psychoactive substance in the US (Fataar and Hammond, 2019), vaping tetrahydrocannabinol (THC) and cannabinol (CBD) (two common constituents of cannabis) is becoming more prevalent among youth and young adults (Miech et al., 2019, Morean et al., 2015, Schulenberg et al., 2020). For example, a cohort study found that the trajectories of nicotine and cannabis vaping frequencies increased similarly over time in youth aging up into adulthood (Lanza et al., 2020). These similarities in use patterns may derive from common risk factors such as EVP marketing/advertising strategies that may promote cannabis vaping in addition to nicotine (Kreitzberg et al., 2019). While studies have evaluated cannabis vaping among adult populations, they often do not consider the possible intersection between nicotine and cannabis vaping (Baldassarri et al., 2020, Braymiller et al., 2020, Pokhrel et al., 2020). In one investigation, vaping nicotine was associated with increased odds of vaping cannabis among a subset of older adults, suggesting that both nicotine and cannabis vaping are important to consider in epidemiological research involving EVP use (Baldassarri et al., 2020).

Concurrent use (co-use) of tobacco and cannabis is common among young adults (Cohn et al., 2018, Lanza et al., 2020), and EVPs provide yet another route of administration for both substances (Lanza et al., 2020, Rabin and George, 2015). Co-use of nicotine and cannabis may cause negative health consequences, such as the development of substance use disorders, additive toxicant exposure, and difficulty quitting both substances (Driezen et al., 2021, Lemyre et al., 2019, Meier and Hatsukami, 2016, Rabin and George, 2015, Ramo et al., 2013, Smith et al., 2020). However, these health effects have only been investigated in the context of combustible tobacco and cannabis co-use. Little is known about the extent to which vaping both substances leads to deleterious health outcomes. While the long-term health consequences of nicotine and cannabis vaping are not well known, nicotine and cannabis vaping have been linked to poor respiratory outcomes (Braymiller et al., 2020, Xie et al., 2020). Thus, it is important to explore use patterns via route of administration for better context into which populations are most susceptible to the health effects of nicotine and cannabis vaping.

In recent years, epidemiological studies on cannabis vaping, alone and in conjunction with nicotine vaping, have gained traction; however, they either tend to focus on youth and young adults or do not consider the extent to which nicotine and cannabis vaping intersect (Lanza et al., 2020, Miech et al., 2019, Morean et al., 2015, Patrick et al., 2020). Thus, we used data from a nationally representative sample of US adults to examine the proportions and sociodemographic correlates of nicotine and cannabis vaping patterns among adults who currently use EVPs (i.e., in the past 30 days). The objective of our study is to better understand the intersecting patterns of nicotine and cannabis vaping among US adults and whether they differ by age, sex, race/ethnicity, sexual orientation status, and socioeconomic status (SES). This study may identify groups that are more susceptible to nicotine and cannabis vaping and inform interventions and policies aimed to reduce nicotine and cannabis consumption.

## 2 Methods

### 2.1 Study sample

We used data from Wave 4 of the Population Assessment of Tobacco and Health (PATH) Study, collected from December 2016 to January 2018, to cross-sectionally examine nicotine and cannabis vaping patterns among adults (18+) who used EVPs in the past 30 days (n = 4082). The PATH Study investigators collected a replenishment sample at Wave 4 to account for attrition and approximate the Wave 1 sample size. Estimates from Wave 4 are nationally representative of the US civilian, noninstitutionalized population. Additional information on the PATH Study sampling design, participant documentation, and data access can be found elsewhere (Hyland et al., 2017). Respondents with missing data on nicotine and cannabis vaping indicators (n = 55) and covariates (n = 232) were excluded, resulting in a final analytic sample of 3795. This research is considered not regulated by the University of Michigan Institutional Review Board due to the use of de-identified, publicly available data.

### 2.2 Measures

#### 2.2.1 Nicotine and cannabis vaping

All PATH Study respondents were asked if they had ever used “electronic nicotine products” (i.e., EVPs) and if they had used EVPs in the past 30 days. Although the question asked about “electronic nicotine products”, we used the term EVPs due to the additional focus on cannabis vaping. We defined current EVP use as adults who used EVPs in the past 30 days. Adults who used EVPs in the past 30 days were asked to indicate if the “electronic nicotine product” they used most often contained nicotine (yes or no). Those who responded “yes” were categorized as adults who vape nicotine, and those who responded “no” were categorized as adults who do not vape nicotine.

Later in the questionnaire, adults who ever used EVPs were asked, “Have you ever used marijuana, marijuana concentrates, marijuana waxes, THC, or hash oils in an electronic product such as an e-cigarette, vape, mod, personal vaporizer, e-hookah, or hookah pen?” with response options “yes” or “no”. Those who indicated “yes” were asked, “when you have used an electronic product, how often were you using it to smoke marijuana, marijuana concentrates, marijuana waxes, THC, or hash oils?” (i.e., never, rarely, sometimes, most of the time, every time). We categorized “rarely” through “every time” as adults who vape cannabis. Respondents who indicated “never” were aggregated with adults who used EVPs and replied “no” to the question about ever vaping cannabis, and categorized as adults who do not vape cannabis.

Based on these four groups, we created a four-category variable for adults who currently vape: 1) nicotine only, 2) cannabis only, 3) nicotine and cannabis, and 4) non-nicotine/non-cannabis e-liquid.

#### 2.2.2 Sociodemographic characteristics

We examined sex (male, female), age (18–24, 25–34, and 35 or older), race/ethnicity (Hispanic, non-Hispanic (NH) White, NH Black, NH Other), sexual orientation status (heterosexual, sexual minority), educational attainment (high school degree/GED or less, some college or more), and annual household income (<$50,000, ≥$50,000) as potential sociodemographic predictors. NH Other included NH American Indian/Alaska Native, NH Asian, and NH multiracial adults. Sexual minority respondents identified themselves as lesbian, gay, bisexual, or “something else” (i.e., LGB+ adults). We dichotomized annual household income to pull in 767 (2.3%) additional respondents who did not answer the original income question but indicated whether their income was either below $50,000 or above $50,000. Supplementary Table 1 displays the overall proportions of adults who currently use EVPs by each sociodemographic characteristic.

#### 2.2.3 Statistical analysis

Proportions and 95% confidence intervals (CI) of nicotine and cannabis vaping among adults who currently use EVPs overall and by sex, age, race/ethnicity, sexual orientation status, educational attainment, and annual household income were calculated. In descriptive analyses, we used Rao-Scott chi-square tests of independence to estimate differences in each sociodemographic characteristics by nicotine and cannabis vaping. We used multinomial logistic regression to examine sociodemographic predictors of each nicotine and cannabis vaping category compared to vaping nicotine only. Further, we conducted sensitivity analyses to evaluate the potential misclassification of adults who vape cannabis by redefining adults who “rarely” vape cannabis as adults who do not vaping cannabis. To account for the complex survey design, we produced weighted estimates and calculated variance using the Balanced Repeated Replication method with Fay’s adjustment set to 0.3 (Judkins, 1990) in Stata 16.1 (StataCorp, 2019).

## 3 Results

Among adults who currently use EVPs, 54.2% vaped nicotine only, 7.4% vaped cannabis only, 23.8% vaped nicotine and cannabis, and 14.6% vaped non-nicotine/non-cannabis e-liquid (Table 1). Most males (51.7%) and females (57.4%) vaped nicotine only, compared to other vaping categories. However, more males vaped nicotine and cannabis (26.5%) than females (20.3%). Patterns of vaping nicotine and cannabis varied by age group, with only 37.3% of young adults aged 18–24 vaping nicotine only, while 52.5% of adults aged 25–34 and 67.0% of adults 35 or older vaped nicotine only. A higher proportion of young adults (10.8%) vaped cannabis only compared to adults aged 25–34 (6.6%) and adults aged 35 or older (5.6%). Approximately 30% of young adults (30.5%) and adults aged 25–34 (28.7%) vaped nicotine and cannabis, which was higher than adults aged 35+ (15.9%). Furthermore, more than 20% of young adults vaped non-nicotine/non-cannabis e-liquid (21.4%), compared to 12.2% of adults aged 25–34 and 11.5% of adults aged 35+.

**Table 1:** Proportions of Nicotine and Cannabis Vaping Among Adults Who Currently Use Electronic Vapor Products (EVPs) Overall and by Sociodemographic Characteristics (N = 3795).

	Proportions of Nicotine and Cannabis Vaping								
	Nicotine Only		Cannabis Only		Nicotine and Cannabis		Non-Nicotine/Non-Cannabis E-liquid		
Sociodemographic characteristics	n	% (95% CI)	n	% (95% CI)	n	% (95% CI)	n	% (95% CI)	P-value^a^
Overall	1889	54.2 (52.1, 56.2)	318	7.4 (6.2, 8.8)	957	23.8 (22.5, 25.2)	631	14.6 (13.3, 16.0)	
Sex									0.013
Female	913	57.4 (54.3, 60.4)	156	7.7 (6.1, 9.8)	383	20.3 (18.1, 22.6)	278	14.6 (12.9, 16.5)	
Male	976	51.7 (48.7, 54.7)	162	7.2 (5.6, 9.2)	574	26.5 (24.1, 29.0)	353	14.6 (12.8, 16.6)	
Age group									<0.001
18–24	625	37.3 (34.8, 39.9)	189	10.8 (8.7, 13.2)	481	30.5 (28.1, 33.1)	378	21.4 (19.4, 23.5)	
25–34	462	52.5 (48.7, 56.3)	73	6.6 (5.1, 8.4)	271	28.7 (25.5, 32.2)	107	12.2 (9.6, 15.5)	
35+	802	67.0 (63.6, 70.2)	56	5.6 (3.6, 8.6)	205	15.9 (13.8, 18.2)	146	11.5 (9.8, 13.5)	
Race/ethnicity									<0.001
Hispanic	246	37.1 (32.9, 41.5)	109	13.5 (10.6, 17.2)	192	25.9 (22.0, 30.2)	165	23.5 (19.3, 28.3)	
NH White	1304	59.8 (57.1, 62.5)	133	5.6 (4.1, 7.6)	602	23.7 (21.9, 25.6)	293	10.8 (9.6, 12.1)	
NH Black	172	45.5 (40.1, 51.1)	50	10.1 (7.2, 14.1)	72	18.1 (14.2, 22.7)	125	26.2 (21.9, 31.1)	
NH Other	167	49.6 (41.5, 57.6)	26	7.3 (4.5, 11.7)	91	28.7 (21.0, 37.9)	48	14.5 (9.8, 21.0)	
Sexual orientation									0.025
Heterosexual	1664	55.1 (52.9, 57.4)	271	7.2 (6.0, 8.7)	791	22.9 (21.3, 24.5)	551	14.7 (13.4, 16.2)	
Lesbian, gay, or bisexual+	225	47.1 (41.6, 52.5)	47	8.7 (5.4, 13.6)	166	30.5 (25.9, 35.6)	80	13.7 (10.7, 17.4)	
Highest educational attainment									0.030
High school or less	901	56.1 (53.3, 58.8)	159	6.3 (5.2, 7.8)	434	22.0 (19.9, 24.3)	348	15.5 (13.9, 17.3)	
Some college or more	988	52.4 (49.5, 55.4)	159	8.4 (6.5, 10.7)	523	25.4 (23.4, 27.6)	283	13.8 (12.0, 15.8)	
Annual household income									0.066
<$50,000	1251	53.2 (50.6, 55.8)	221	7.0 (5.9, 8.3)	635	23.7 (22.0, 25.5)	458	16.1 (14.4, 18.0)	
$50,000+	638	56.0 (52.4, 59.4)	97	8.2 (5.7, 11.6)	322	24.0 (21.5, 26.7)	173	11.8 (10.0, 14.0)	

Data come from Wave 4 of the Population Assessment of Tobacco and Health (PATH) Study.

a Rao-Scott chi-square test of independence comparing proportions of each sociodemographic characteristic by nicotine and cannabis vaping.

Vaping patterns also differed by other sociodemographic characteristics. More NH White adults vaped nicotine only (59.8%) compared to NH Other (49.6%), NH Black (45.5%), and Hispanic adults (37.1%). A higher proportion of Hispanic adults (13.5%) vaped cannabis only compared to NH White adults (5.6%). Furthermore, more Hispanic (23.5%) and NH Black (26.2%) vaped non-nicotine/non-cannabis e-liquid compared to their NH White counterparts (10.8%). Nicotine and cannabis vaping varied by sexual orientation status with heterosexual adults (55.1%) having higher proportions of nicotine only vaping, compared to LGB+ adults (47.1%). However, more LGB+ adults (30.5%) vaped nicotine and cannabis compared to heterosexual adults (22.9%). We observed minimal differences in nicotine and cannabis vaping by SES, with more adults with an annual household income of <$50,000 (16.1%) vaping non-nicotine/non-cannabis e-liquid, compared to adults with an annual household income of $50,000 or more (11.8%).

In the adjusted multinomial logistic regression models (Table 2), young adults (aged 18–24) (vs. adults aged 35 or older) had at least three-fold greater odds of vaping cannabis only (OR: 3.19, 95% CI: 1.89–5.39), vaping nicotine and cannabis (OR: 3.13, 95% CI: 2.46–3.98), and vaping non-nicotine/non-cannabis e-liquid (OR: 3.18, 95% CI: 2.50–4.01), compared to vaping nicotine only. In addition, adults aged 25–34 (vs. adults aged 35 or older) had two times greater odds of vaping nicotine and cannabis (OR: 2.08, 95% CI: 1.63–2.65), compared to vaping nicotine only. Hispanic (vs. NH White) adults had at least three times greater odds of vaping cannabis only (OR: 3.73, 95% CI: 2.47–5.61) and vaping non-nicotine/non-cannabis e-liquid (OR: 3.11, 95% CI: 2.24–4.33), and 1.5 times greater odds of vaping nicotine and cannabis (OR: 1.50, 95% CI: 1.11–2.01), compared to vaping nicotine only. Similarly, NH Black (vs. NH White) adults had 2.5 times greater odds of vaping cannabis only (OR: 2.59, 95% CI: 1.55–4.31) and at least three times greater odds of vaping non-nicotine/non-cannabis e-liquid (OR: 3.23, 95% CI: 2.34–4.46), compared to vaping nicotine only. Furthermore, LGB+ (vs. heterosexual) adults had 1.5 times greater odds of vaping nicotine and cannabis (OR: 1.51, 95% CI: 1.12–2.04), compared to vaping nicotine only. Adults who had a high school education or less (vs. some college or more) had 20% lower odds (OR: 0.80, 95% CI: 0.65–0.98) of vaping nicotine and cannabis, compared to those who only vaped nicotine.

**Table 2:** Multivariable Multinomial Logistic Regression Models of Associations Between Sociodemographic Characteristics and Proportions of Nicotine and Cannabis Vaping Among Adults Who Currently Use Electronic Vapor Products (EVPs) (n = 3795).

	Proportions of Nicotine and Cannabis Vaping^a^					
	Cannabis Only		Nicotine & Cannabis		Non-Nicotine/Non-Cannabis E-liquid	
Sociodemographic Characteristics	AOR^b^	95% CI	AOR^b^	95% CI	AOR^b^	95% CI
Sex						
Female	1.11	0.75, 1.65	0.72	0.57, 0.92	1.04	0.79, 1.37
Male	REF		REF		REF	
Age group						
18–24	**3.19**	**1.89, 5.39**	**3.13**	**2.46, 3.98**	**3.18**	**2.50, 4.01**
25–34	1.30	0.74, 2.27	**2.08**	**1.63, 2.65**	1.20	0.84, 1.71
35+	REF		REF		REF	
Race/ethnicity						
Hispanic	**3.73**	**2.47, 5.61**	**1.50**	**1.11, 2.01**	**3.11**	**2.24, 4.33**
NH White	REF		REF		REF	
NH Black	**2.59**	**1.55, 4.31**	0.99	0.67, 1.45	**3.23**	**2.34, 4.46**
NH Other	1.38	0.67, 2.85	1.29	0.80, 2.07	1.50	0.94, 2.40
Sexual orientation						
Heterosexual	REF		REF		REF	
Lesbian, gay, or bisexual+	1.18	0.70, 1.98	**1.51**	**1.12, 2.04**	0.89	0.63, 1.26
Highest educational attainment						
High school or less	0.70	0.50, 1.00	**0.80**	**0.65, 0.98**	0.97	0.77, 1.23
Some college or more	REF		REF		REF	
Annual household income						
<$50,000	0.80	0.54, 1.19	1.07	0.87, 1.31	1.22	0.91, 1.65
$50,000+	REF		REF		REF	

Data come from Wave 4 of the Population Assessment of Tobacco and Health (PATH) Study.

Bold values denote statistical significance (p < 0.05). AOR: adjusted odds ratio.

a The outcome referent group: vaping nicotine only.

b Odds ratios and 95% confidence intervals were adjusted for all sociodemographic characteristics.

As expected, our sensitivity analyses reclassifying adults who only vape cannabis rarely as adults who do not vape cannabis resulted in lower proportions of cannabis only (5.3%) or nicotine and cannabis vaping (13.2%) (Supplementary Table 2). Results from regression models remained similar in statistical significance and magnitude, except an association involving highest educational attainment (Supplementary Table 3).

## 4 Discussion

Our study found that more than half (54.2%) of adults who used EVPs indicated that they vaped nicotine only, while about a quarter (23.8%) vaped nicotine and cannabis, 14.6% vaped non-nicotine/non-cannabis e-liquid, and 7.4% vaped cannabis only. Results from our regression model indicated that nicotine and cannabis vaping patterns differed by age, race/ethnicity, sexual orientation, and educational attainment. Notably, young adults (versus older adults), Hispanic and NH Black (vs NH White) adults, and LGB+ (vs heterosexual) adults had higher odds of vaping either cannabis only, nicotine and cannabis, or non-nicotine/non-cannabis e-liquid compared to vaping nicotine only. These findings collectively call for further investigation on the intersection of nicotine and cannabis vaping to better understand the population prevalence of use among adults.

Most prior research on nicotine and cannabis vaping focuses on younger populations, with limited investigations of adult samples (Baldassarri et al., 2020, Braymiller et al., 2020, Liautaud et al., 2021, Steigerwald et al., 2018). One study found that the prevalence of cannabis vaping was more common among young adults aged 18–34 years compared to adults aged 50 years or older (Steigerwald et al., 2018). In our study, young adults had greater odds of vaping cannabis only, vaping nicotine and cannabis together, and vaping non-nicotine/non-cannabis e-liquid, compared to vaping nicotine only. In addition, adults aged 25–34 had greater odds of vaping nicotine and cannabis, compared to vaping nicotine only. These findings emphasize the importance of addressing not only nicotine vaping among younger adult populations, but also vaping other substances such as cannabis and perhaps additives that do not include nicotine or cannabis such as flavorings (Miech et al., 2017).

Regarding differences by race/ethnicity, we found that Hispanic adults had greater odds of vaping cannabis only, nicotine and cannabis, and non-nicotine/non-cannabis e-liquid versus nicotine only vaping, while NH Black adults had greater odds of vaping cannabis only and non-nicotine/non-cannabis e-liquid versus nicotine only vaping, compared to their NH White counterparts. These results suggest that Hispanic adults are not using EVPs to consume nicotine only, and an important subset (around 10%) of both Hispanic and NH Black adults may be using EVPs to consume cannabis alone. A recent study found that Hispanic and NH Black youth had the highest prevalence of cannabis use with EVPs compared to other racial/ethnic groups (Watson et al., 2021). Factors influencing cannabis vaping may include perceptions of harms or benefits relative to other modes of cannabis delivery, curiosity, or patterns of nicotine use and cannabis smoking unique to certain racial/ethnic groups (e.g., blunting is more common among Hispanic and NH Black populations) (Mantey et al., 2021, Montgomery and Mantey, 2018, Watson et al., 2021). Furthermore, cannabis vaping may bypass the social consequences of cannabis smoking (Smith et al., 2021), which might contribute to the differences we observed in cannabis-only vaping by race/ethnicity.

Research has found that potential health consequences of smoking/vaping cannabis only may be unique from vaping nicotine and could therefore disproportionately impact these populations (Braymiller et al., 2020, King et al., 2020). However, further work that aims to clarify the health effects of vaping cannabis and smoking cannabis, separately, is needed, as studies have revealed that many people who vape cannabis also smoke cannabis (Lee et al., 2016). In addition, the finding that Hispanic and NH Black adults may be vaping non-nicotine/non-cannabis e-liquid more than their NH White counterparts suggests that these consumers may be seeking out vaping flavorings with low to no nicotine or THC content (Miech et al., 2017). The possibility also exists that some adults who currently use EVPs are unaware of the substance contents in their EVPs (Buettner-Schmidt et al., 2021, Raymond et al., 2018), and the non-nicotine/non-cannabis e-liquid use group may be consuming nicotine and/or THC without realizing it.

LGB+ adults had higher odds of vaping nicotine and cannabis relative to vaping nicotine only, compared to heterosexual adults. This finding is somewhat consistent with a previous study indicating that bisexual adults had higher odds of nicotine and cannabis consumption (smoking or vaping), compared to heterosexual adults (Liautaud et al., 2021). Our results, in conjunction with other studies, emphasize a need to address nicotine and cannabis vaping among LGB+ adults to prevent the potential joint health consequences of nicotine and cannabis intake and promote health equity within the LGBTQ community.

While associations involving educational attainment and annual household income were limited, one study among a sample of young adults found that the prevalence of cannabis vaping was more common among respondents with high SES compared to respondents with low SES (Jones et al., 2016). Since our study examined proportions of nicotine and cannabis vaping among adults who currently used EVPs, it is difficult to compare our results directly to other studies that examine nicotine and cannabis vaping separately at the population level. Nevertheless, our results indicate that an association may exist where adults with lower education have lower odds of vaping nicotine and cannabis, relative to vaping nicotine only, compared to adults with higher education, or that adults with higher education vape a mix of nicotine and cannabis relative to adults with lower education.

Cannabis smoking is associated with tobacco cessation difficulty, tobacco use relapse, and increased intensity of tobacco use (Driezen et al., 2021, Rabin and George, 2015). If cannabis vaping has a similar effect, it may undermine cessation efforts for adults who use EVPs. The reverse gateway theory posits that cannabis use may encourage tobacco use, especially if routes of administration are similar (e.g., smoking, vaping) (Lemyre et al., 2019, Rabin and George, 2015). However, drivers of cannabis vaping initiation among those who vape nicotine might differ from drivers of nicotine vaping initiation among those who vape cannabis (Lee et al., 2016, Smith et al., 2021). Adults who use both nicotine and cannabis have reported the heightened subjective effects of cannabis from simultaneous nicotine and cannabis vaping as a primary reason behind co-use (Smith et al., 2021). The extent to which simultaneous nicotine and cannabis vaping varies from simultaneous nicotine and cannabis smoking among adults is not fully understood. Further research is needed to better understand the intersection between cannabis vaping and cannabis smoking, including how mode of cannabis consumption might impact tobacco use outcomes (Fataar and Hammond, 2019, Lee et al., 2016). Nevertheless, the neurobiological effects of cannabis vaping or smoking can complicate the advancement of tobacco regulatory science and may promote nicotine vaping. In 2009, the FDA was granted regulatory authority over tobacco products in the US but does not have direct power over cannabis constituents in e-liquids (Deyton, 2011, Mead, 2019). Understanding nicotine and cannabis vaping patterns is vital to inform tobacco control researchers of the potential implications related to tobacco regulation and cessation efforts.

### 4.1 Limitations

We acknowledge limitations of our study findings. First, due to how the cannabis vaping measures were assessed in the PATH Study (i.e., among nicotine vapers only), it is possible that we missed additional exclusive cannabis vapers in this analysis. In addition, this pattern of assessment may be prone to having adults contradict themselves, such as indicating that they ever vape cannabis but “never” in terms of how frequently they vape the substance, making it difficult to infer which use groups they belong to. Second, it difficult to measure the frequency and intensity by which specific substances are consumed in the PATH Study (Pearson and Villanti, 2020). While respondents were asked about how often they vaped cannabis when using EVPs, we were unable to quantify frequency of cannabis vaping (e.g., by number of days in the past 30 days). Third, cannabis vaping was examined in the context of ever use only, and respondents who currently used EVPs endorsed cannabis vaping may have only vaped cannabis in some period that preceded the past 30 days. Fourth, we simplified the nicotine and cannabis vaping groups in terms of how often they vaped both substances (i.e., vape nicotine most of the time; vape cannabis rarely, sometimes, most of the time, every time). Nevertheless, our non-nicotine-exclusive vaping categories represent varying vaping use patterns with important regulatory and health implications. Fifth, nicotine/cannabis vaping categories relied on self-reports and did not include biochemical verification for all respondents (Hyland et al., 2017). Sixth, due to limited sample sizes, we had to aggregate American Indian/Alaska Native, Asian, and multiracial adults into an NH Other category, and lesbian, gay, and bisexual adults into an LGB+ category. We were additionally unable to disaggregate Hispanic ethnic identity with the present data, which may mask important differences for ethnic subgroups such as Mexican Americans or Puerto Ricans. Finally, data were collected from late 2016 to early 2018, and results from this study may not represent more recent use patterns, particularly in light of the COVID-19 pandemic.

Despite these limitations, our findings demonstrate that the proportion of nicotine and cannabis vaping differs across adult sociodemographic groups, calling for additional work that aims to unveil and address these use disparities. With cannabis vaping on the rise among younger populations (Miech et al., 2019, Schulenberg et al., 2020), efforts aimed at reducing nicotine and cannabis consumption overall and within high-risk groups are needed, especially those that address vaping as an additional route of administration. Our study also examines patterns of nicotine and cannabis vaping, with important variation by how adults vape each substance. These results are presented compared to adults who vape only nicotine to capture differences by use groups that are less of a focus in the substance use literature, particularly the tobacco control literature. Given that one of the larger concerns surrounding EVP use is nicotine intake, presenting groups that vape nicotine in various ways with and without cannabis may shed light on profiles of adults who are at higher risk for downstream health consequences associated with vaping both substances. Another concern involves the health implications for adults who indicate that they vape non-nicotine/non-cannabis e-liquid, warranting further investigation into this group.

## 5 Conclusions

This study aimed to examine sociodemographic differences in patterns of nicotine and cannabis vaping among a nationally representative sample of US adults who currently use EVPs. We found substantial overlap in nicotine and cannabis vaping and important differences in patterns by sociodemographic groups, including age, race/ethnicity, sexual orientation status, and educational attainment. Our findings imply that an important proportion of adults who currently used EVPs may be susceptible to the health effects of both nicotine and cannabis. To improve public health, prevention and cessation interventions aimed at reducing nicotine intake must recognize that a large proportion of adults who currently use EVPs may be vaping cannabis alone or in addition to nicotine.

Research reported in this publication was supported by the National Cancer Institute of the National Institutes of Health (NIH) and FDA Center for Tobacco Products (CTP) under Award Number U54CA229974. The content is solely the responsibility of the authors and does not necessarily represent the official views of the NIH or the Food and Drug Administration.

## CRediT authorship contribution statement

**Delvon T. Mattingly:** Conceptualization, Methodology, Formal analysis, Writing – original draft, Writing – review & editing, Visualization. **Akash Patel:** Formal analysis, Writing – review & editing. **Jana L. Hirschtick:** Writing – review & editing. **Nancy L. Fleischer:** Conceptualization, Writing – review & editing, Supervision, Funding acquisition.

## Declaration of Competing Interest

The authors declare that they have no known competing financial interests or personal relationships that could have appeared to influence the work reported in this paper.
