# Supplementary material for: Sociodemographic differences in patterns of nicotine and cannabis vaping among US adults
Source: Prev Med Rep. 2022 Jan 27;26:101715. doi: 10.1016/j.pmedr.2022.101715 (PMC8814645; doi:10.1016/j.pmedr.2022.101715)
Supplement: Supplementary data 1 [file mmc1.docx]

| **Supplementary Table 1. Proportions of Participant Characteristics Among Adults Who Currently Use Electronic Vapor Products (EVPs) (N=3,795)** | | |
| --- | --- | --- |
|  | n | % (95% CI) |
| Sex |  |  |
| Female | 1730 | 42.7 (40.7, 44.6) |
| Male | 2065 | 57.3 (55.4, 59.3) |
| Age group |  |  |
| 18-24 | 1673 | 29.5 (28.0, 31.1) |
| 25-34 | 913 | 28.2 (26.4, 30.0) |
| 35+ | 1209 | 42.3 (40.4, 44.3) |
| Race/ethnicity |  |  |
| Hispanic | 712 | 15.0 (13.6, 16.4) |
| NH White | 2332 | 67.0 (65.0, 69.0) |
| NH Black | 419 | 10.7 (9.5, 12.0) |
| NH Other | 332 | 7.4 (6.4, 8.5) |
| Sexual orientation |  |  |
| Heterosexual | 3277 | 87.8 (86.2, 89.1) |
| Lesbian, gay, or bisexual+ | 518 | 12.2 (10.9, 13.8) |
| Highest educational attainment |  |  |
| High school or less | 1842 | 47.0 (44.7, 49.2) |
| Some college or more | 1953 | 53.0 (50.8, 55.3) |
| Annual household income |  |  |
| <$50,000 | 2565 | 65.3 (63.1, 67.4) |
| $50,000+ | 1230 | 34.7 (32.6, 36.9) |
| Data come from Wave 4 of the Population Assessment of Tobacco and Health (PATH) Study | | |

| **Supplementary Table 2. Change in Proportions of Nicotine and Cannabis Vaping Among Adults Who Currently Use Electronic Vapor Products (EVPs) With Varying Definitions for Cannabis Vaping (N=3,795)** | | |
| --- | --- | --- |
|  | n | % (95% CI) |
| Nicotine and cannabis vaping (original definition) ^a^ |  |  |
| Nicotine only | 1889 | 54.2 (52.1, 56.2) |
| Cannabis only | 318 | 7.4 (6.2, 8.8) |
| Nicotine and cannabis | 957 | 23.8 (22.5, 25.2) |
| Non-nicotine and non-cannabis e-liquid | 631 | 14.6 (13.3, 16.0) |
| Nicotine and cannabis vaping (sensitivity analysis) ^b^ |  |  |
| Nicotine only | 2304 | 64.7 (62.7, 66.7) |
| Cannabis only | 219 | 5.3 (4.2, 6.7) |
| Nicotine and cannabis | 542 | 13.2 (12.0, 14.6) |
| Non-nicotine and non-cannabis e-liquid | 730 | 16.7 (15.3, 18.2) |
| Data come from Wave 4 of the Population Assessment of Tobacco and Health (PATH) Study | | |
| ^a^ "Rarely" cannabis use is defined as having used cannabis |  |  |
| ^b^ "Rarely" cannabis use is defined as not having used cannabis |  |  |

| **Supplementary Table 3. Multivariable Multinomial Logistic Regression Models of Associations Between Participant Sociodemographic Characteristics and Proportions of Nicotine and Cannabis Vaping Among Adults Who Currently Use Electronic Vapor Products (EVPs) (n=3,795)** | | | | | | |
| --- | --- | --- | --- | --- | --- | --- |
|  | Proportions of Nicotine and Cannabis Vaping^a, b^ | | | | | |
|  | Cannabis Only | | Nicotine & Cannabis | | Non-Nicotine/Non-Cannabis E-liquid | |
| Characteristics | AOR^c^ | 95% CI | AOR^c^ | 95% CI | AOR^c^ | 95% CI |
| Sex |  |  |  |  |  |  |
| Female | 1.10 | 0.66, 1.84 | 0.77 | 0.59, 1.00 | 1.13 | 0.89, 1.45 |
| Male | REF |  | REF |  | REF |  |
| Age group |  |  |  |  |  |  |
| 18-24 | **2.50** | **1.26, 4.98** | **3.03** | **2.28, 4.02** | **2.73** | **2.19, 3.40** |
| 25-34 | 1.16 | 0.57, 2.36 | **2.29** | **1.69, 3.10** | 1.14 | 0.82, 1.58 |
| 35+ | REF |  | REF |  | REF |  |
| Race/ethnicity |  |  |  |  |  |  |
| Hispanic | **3.36** | **2.06, 5.49** | **1.49** | **1.07, 2.06** | **3.02** | **2.26, 4.04** |
| NH White | REF |  | REF |  | REF |  |
| NH Black | **2.56** | **1.46, 4.50** | 1.09 | 0.71, 1.66 | **3.24** | **2.40, 4.39** |
| NH Other | 1.32 | 0.56, 3.11 | 1.52 | 0.87, 2.67 | 1.50 | 0.97, 2.34 |
| Sexual orientation |  |  |  |  |  |  |
| Heterosexual | REF |  | REF |  | REF |  |
| Lesbian, gay, or bisexual+ | 1.29 | 0.71, 2.33 | **1.54** | **1.12, 2.10** | 0.83 | 0.60, 1.13 |
| Highest educational attainment |  |  |  |  |  |  |
| High school or less | 0.71 | 0.47, 1.05 | 0.88 | 0.71, 1.10 | 1.00 | 0.82, 1.23 |
| Some college or more | REF |  | REF |  | REF |  |
| Annual household income |  |  |  |  |  |  |
| <$50,000 | 0.90 | 0.55, 1.48 | 1.23 | 0.98, 1.54 | 1.13 | 0.88, 1.47 |
| $50,000+ | REF |  | REF |  | REF |  |
| Data come from Wave 4 of the Population Assessment of Tobacco and Health (PATH) Study | | | | | | |
| Bold values denote statistical significance (p<0.05) | | | | | | |
| ^a^ The outcome referent group: vaping nicotine only | | | | | | |
| ^b^ "Rarely" cannabis use is defined as not having used cannabis | | | | | | |
| ^c^ Adjusted odds ratios (AOR) and 95% confidence intervals adjusted for all sociodemographic characteristics | | | | | | |
